# Paying Upfront: Successful Initial Infections Protect Against Severe Future Infections

**DOI:** 10.1093/iob/obag050

**Published:** 2026-08-21

**Authors:** K M Talbott, A E Fleming-Davies, A A Pérez-Umphrey, A E Henschen, J Garrett-Larsen, F E Tillman, J N Weil, A G Arneson, S J Geary, E R Tulman, L M Childs, K E Langwig, D M Hawley, J S Adelman

**Affiliations:** Department of Biological Sciences, University of Memphis, Memphis, TN 38152, USA; Department of Biology, University of San Diego, San Diego, CA 92110, USA; Department of Biology, University of San Diego, San Diego, CA 92110, USA; Department of Biological Sciences, Virginia Tech, Blacksburg, VA 24061, USA; Department of Biological Sciences, University of Memphis, Memphis, TN 38152, USA; Department of Biological Sciences, Eastern Illinois University, Charleston, IL 61920, USA; Department of Biological Sciences, Virginia Tech, Blacksburg, VA 24061, USA; Department of Biological Sciences, University of Memphis, Memphis, TN 38152, USA; Department of Biological Sciences, University of Memphis, Memphis, TN 38152, USA; Department of Biological Sciences, Virginia Tech, Blacksburg, VA 24061, USA; Department of Pathobiology and Veterinary Science, University of Connecticut, Storrs, CT 06269, USA; Department of Pathobiology and Veterinary Science, University of Connecticut, Storrs, CT 06269, USA; Department of Mathematics, Virginia Tech, Blacksburg, VA 24061, USA; Department of Biological Sciences, Virginia Tech, Blacksburg, VA 24061, USA; Department of Biological Sciences, Virginia Tech, Blacksburg, VA 24061, USA; Department of Biological Sciences, University of Memphis, Memphis, TN 38152, USA

## Abstract

Understanding the consistency with which individual hosts respond to repeated pathogen exposures is crucial for accurately modeling pathogen transmission and eco-evolutionary dynamics. When vertebrate hosts face repeated pathogen exposures, immune memory typically reduces the probability and/or severity of subsequent infections, yet it remains unclear whether individual hosts remain consistent in their level of response relative to other individuals. We investigated this question in house finches (*Haemorhous mexicanus*) from two populations varying in their history of endemism of the bacterial pathogen *Mycoplasma gallisepticum* (MG). MG-naïve individuals were experimentally inoculated twice with MG, allowing recovery between inoculations. We then asked if individual host’s responses (i.e., susceptibility, resistance, and tolerance) to the second inoculation were predicted by their responses to initial inoculation, population of origin, or sex. Our results suggest that individuals were not consistent in their relative response to repeated exposure, although individuals that were relatively tolerant to initial MG infection had reduced probability of subsequent infection. Compared to finches successfully infected following first MG exposure, those that were uninfected following their first MG exposure were more likely to be infected upon subsequent exposure. Furthermore, these infections were more severe, with higher pathogen loads and reduced tolerance in finches that were unsusceptible to their first MG exposure. Demographic factors were important predictors of susceptibility, but not tolerance or resistance, to a second MG exposure. Finches from the MG-endemic population and males were less susceptible to second MG exposure than finches from the MG-naïve population and females, respectively. Incorporating individual variation in response to subsequent exposures can shed light on transmission dynamics and the evolution of host defense strategies in systems characterized by reinfections.

## Abstract (es)

Comprender la consistencia con la que huéspedes individuales responden a la exposición repetida a un patógeno es crucial para poder crear un modelo que con precisión refleje la transmisión de dicho patógeno y dinámicas eco-evolucionarias. Cuando huéspedes vertebrados se confrontan a la exposición repetida a un patógeno, la memoria inmunológica típicamente reduce la probabilidad y/severidad de infecciones subsecuentes, aunque no es claro si huéspedes individuales son consistentes en su nivel de respuesta en relación a otros individuos. Investigamos esta pregunta en pinzones domésticos (*Hamorhous mexicanus*), comparando dos grupos diferentes en la que varía su historia endémica a la bacteria patógena *Mycoplasma gallisecticum* (MG). Individuos jamás expuestos a MG fueron inoculados dos veces con este patógeno permitiendo recuperación entre inoculaciones. A este punto, nos preguntamos si la respuesta de cada huésped (i.e., susceptibilidad, resistencia, tolerancia) a la segunda inoculación era predecible por su reacción a la inoculación inicial, población de origen, o sexo. Nuestros resultados sugieren que los individuos no fueron consistentes en su respuesta relativa a una repetida exposición al patógeno, aunque individuos que fueron relativamente tolerantes a la infección inicial con MG mostraron una probabilidad reducida a infección subsecuente. Aún más, las infecciones fueron más severas, con cargas patógenas más altas y tolerancia reducida, en pinzones que fueron insensibles a su primera exposición. Factores demográficos fueron importantes en predecir susceptibilidad, pero no tolerancia o resistencia a una segunda exposición de MG. Pinzones machos pertenecientes a una población MG endémica, fueron menos susceptibles a una segunda exposición al patógeno en comparación a una población de hembras y sin exposición previa a MG respectivamente. Incorporando variaciones individuales en la respuesta a exposiciones subsecuentes, puede arrojar luz sobre la dinámicas de transmisión y sobre la evolución de estrategias de defensa de los huéspedes en sistemas caracterizados por reinfecciones.

## Introduction

At both individual and population levels, hosts show considerable heterogeneity in their responses to pathogens. One potentially critical and often overlooked driver of variation is prior exposure to a given pathogen (Leon and Hawley 2017; Hawley et al. 2024; Vale et al. 2025). While relevant empirical studies tend to focus on single pathogen exposures, hosts in a natural context often face repeated exposures to the same pathogen, as demonstrated by reinfections in humans and wildlife (Frank and Taber 1983; Quinnell et al. 2010; Trottier et al. 2010; Lier et al. 2014; Müller‐Klein et al. 2019; Dos Santos et al. 2021). While acquired immunity generally reduces the severity of subsequent infections, it is also possible that individual variation in responses to initial exposure, such as the severity of initial infection, affects responses to subsequent exposures. Additional work is needed to understand the relative consistency of individual host responses to infection, which is important not only for improving epidemiological models, but also for better understanding the physiological mechanisms and costs associated with those responses.

Host responses are often characterized in terms of susceptibility, resistance, and tolerance, all of which can vary within and between populations (Table 1). Here, we define host susceptibility as the individual probability of infection following pathogen exposure, based on a binomial variable of “infected” or “uninfected” (Hawley et al. 2024). Resistance is defined as the ability of the host to reduce pathogen replication during infection, with more-resistant individuals showing lower pathogen loads than less-resistant conspecifics (Gandon and Michalakis 2000; Råberg et al. 2007, 2009). Tolerance is defined as the per-pathogen impact of infection on host fitness, with more-tolerant hosts showing smaller reductions in fitness at the same pathogen burden as less-tolerant hosts (Caldwell et al. 1958; Råberg et al. 2009; Little et al. 2010; Adelman et al. 2013; Kutzer and Armitage 2016). A commonly used proxy for quantifying fitness reductions caused by infection is host pathology, which can reflect damage caused by pathogen replication and/or host responses to the pathogen (i.e., immunopathology) and is negatively correlated with host fitness in general (Råberg et al. 2009).

**Table 1:** Definitions of host responses to parasitism.

Term	Definition
Susceptibility	Probability of infection in an individual host following pathogen exposure at a given dose, based on a binomial variable of “infected” or “uninfected”
Resistance	Ability of the host to regulate within-host pathogen growth, quantified as pathogen load; individual hosts with higher resistance have relatively lower pathogen loads
Tolerance	Per-pathogen impact of infection on host fitness; here, pathology is used as a proxy for fitness, with more tolerant individuals experiencing less pathology per unit of pathogen than less tolerant individuals

Investigating individual consistency in host responses in terms of susceptibility, tolerance, and resistance is key to refining predictions about epidemiological and host-pathogen coevolutionary dynamics. For example, it is often assumed that individuals show consistency in susceptibility, meaning that individuals that are not successfully infected upon initial pathogen exposure are also unlikely to be infected upon subsequent exposure; however, this may not be the case. Similarly, if individual resistance to initial exposure is predictive of resistance to subsequent exposure, incorporating this dynamic may elucidate shifts in individual competence over time because higher resistance necessarily decreases an individual’s ability to infect other potential hosts (i.e., host competence; (Martin et al. 2019)). There is a similar need to understand consistency in tolerance, as host tolerance may have distinct impacts on host competence, depending on the mode of pathogen transmission and effects of infection on host behavior (Lloyd-Smith et al. 2005; Adelman and Hawley 2017; Burgan et al. 2019; Ruden and Adelman 2021; Henschen et al. 2025). Furthermore, individual shifts in tolerance and resistance may each have different ramifications for host population recovery following the emergence of novel pathogens (Wilber et al. 2024), as well as for the evolution of pathogen virulence (i.e., harm caused to the host (Pfennig 2001; Miller et al. 2005)).

In systems where repeated exposure to the same pathogen is common, the predicted relationship between individual responses to initial and subsequent exposures can depend on the type of host response examined, as well as the immune mechanisms involved. Vertebrate host responses to initial exposure depend largely on the innate immune response, a hallmark of which is inflammation (Janeway 2001; Ashley et al. 2012). In addition to directly killing invading pathogens, activated innate immune cells produce proinflammatory cytokines that help stimulate the subsequent development of an acquired immune response (Beutler 2004). Then, acquired immunity functions to reduce host susceptibility to a second exposure, or failing that, to help reduce pathogen burden (e.g., increase resistance) during a second infection (Janeway 2001). Because robust proinflammatory responses can incur damage to a host’s own tissues (e.g., immunopathology), muting inflammation has been proposed as a potential mechanism of tolerance (Råberg et al. 2009; Sears et al. 2011; Medzhitov et al. 2012; Adelman et al. 2013; Adelman and Hawley 2017). However, it remains unclear whether this muting of inflammation during an initial infection reduces the development of acquired immunity, potentially increasing susceptibility or decreasing resistance to future pathogen exposures (Cressler and Adelman 2024).

**Fig. 1:**
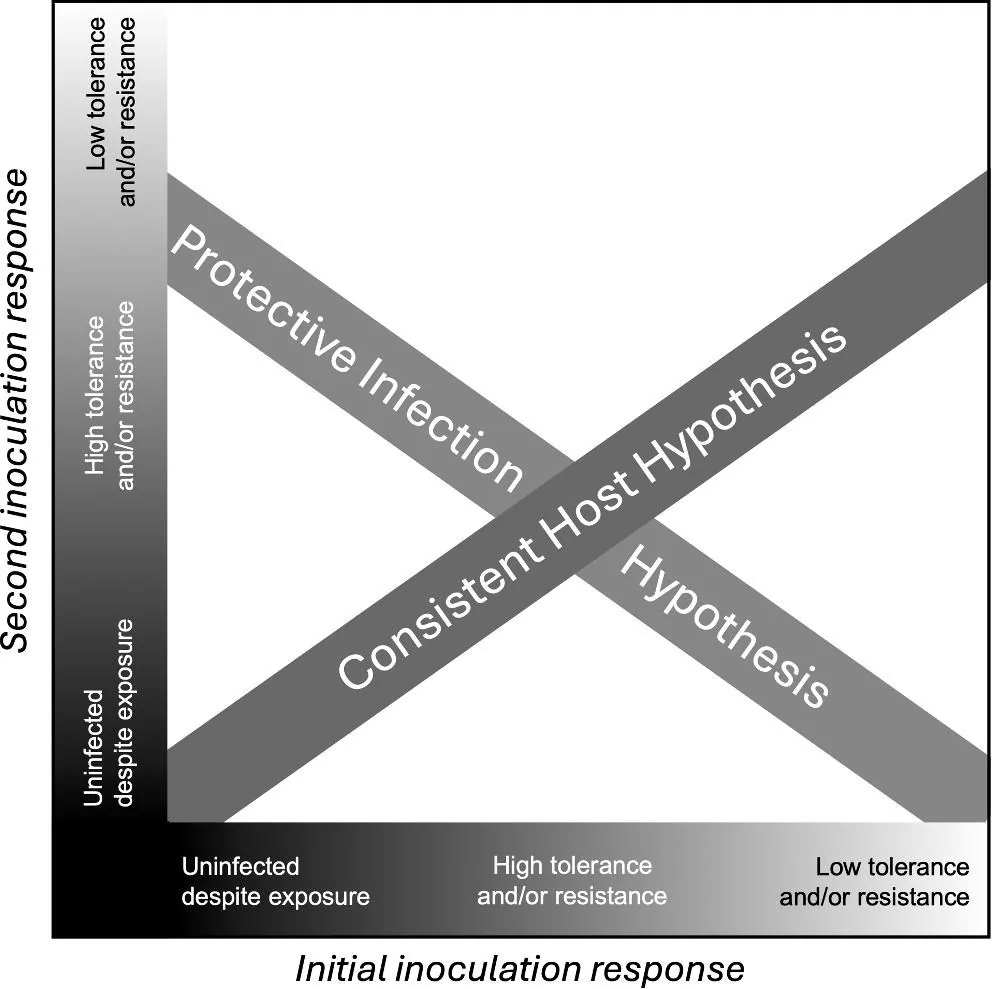
Hypothesized relationships between initial and second inoculation responses in individual house finches (*Haemorhous mexicanus*) inoculated with *Mycoplasma gallisepticum* (MG) bacteria twice. Note that responses form a gradient of infection severity, not discrete response categories, and that the “uninfected” group represents individuals that are exposed to MG but do not show evidence of infection (i.e., detectable load and/or eye score). Prediction line slopes are shallower than a perfect regression to reflect the effect of acquired immune responses to the initial inoculation on responses to the second inoculation (i.e., that all finches successfully infected following initial inoculation should gain some level of protection).

Despite the costs of immunopathology, theory predicts that selection may favor more pronounced host inflammatory responses if they are associated with reduced susceptibility to a second infection (Cressler and Adelman 2024), which we term the “Protective Infection Hypothesis.” The Protective Infection Hypothesis predicts that hosts susceptible to an initial infection, especially those that become the sickest (i.e., low tolerance and/or resistance), would experience either decreased susceptibility to a second exposure or less severe illness during second infection (i.e., high tolerance and/or resistance; see Fig. 1). Similarly, individuals that are not susceptible or show high tolerance and/or resistance to the first inoculation would become the sickest after the second inoculation. Alternatively, some individuals may be consistently less or more resistant, and/or less or more tolerant, relative to others. We term this the “Consistent Host Hypothesis.” Under the Consistent Host Hypothesis, we would predict that individuals who become infected and are the sickest (i.e., higher susceptibility, lower tolerance, and/or lower resistance) following initial inoculation would remain susceptible and become the sickest after a second inoculation, and vice versa for those not susceptible and/or having the least severe responses to initial inoculation. Thus, for example, we expect tolerance during the first and second inoculations to be positively correlated under the Consistent Host Hypothesis but negatively correlated under the Protective Infection Hypothesis (Fig. 1).

To test these hypotheses, we used house finches (*Haemorhous mexicanus*, “finches”) infected with the bacterium *Mycoplasma gallisepticum* (MG) as a model host-pathogen system. MG is a pathogen of poultry that spilled over into wild finches in eastern North America in the mid-1990’s and rapidly spread westward (Ley et al. 1996; Hochachka and Dhondt 2000). In finches, MG can cause severe conjunctivitis, rhinitis, and lethargy (Kollias et al. 2004), which led to finch population declines immediately following spillover (Dhondt et al. 1998; Hochachka and Dhondt 2000; Faustino et al. 2004). MG continues to circulate in finch populations where it has become endemic and experimental studies using multiple MG inoculations illustrate that finches are susceptible to reinfection (Sydenstricker et al. 2005; Leon and Hawley 2017). The severity of subsequent infections may vary by the identity and dose of the initial and subsequent strains (Dhondt et al. 2017), with more-virulent strains (Fleming-Davies et al. 2018) and higher initial doses (Leon and Hawley 2017; Weitzman et al. 2022) providing better protection against subsequent infection than less-virulent strains and lower initial doses. In addition, recent work has shown that MG exposure reduces mean susceptibility and increases heterogeneity in susceptibility to a second inoculation among finches from an MG-endemic population (Hawley et al. 2024). This indicates that while individuals gain some protection against a second infection from initial exposure, the extent of this protection varies. Despite the consequences of this variation for eco-evolutionary dynamics, factors driving variation in protection at the individual level (e.g., population origin, sex) remain understudied, and whether responses to initial and subsequent exposures are consistent within individuals, has not yet been examined.

Demographic variation in host responses to initial pathogen exposure are well documented. For example, host populations may evolve tolerance, resistance, and/or reduced susceptibility to emerging pathogens (Bonneaud et al. 2011; Kerr 2012; Atkinson et al. 2013; Savage and Zamudio 2016; Langwig et al. 2017; Voyles et al. 2018; Henschen et al. 2023), resulting in inter-population variation in host responses based on pathogen endemism. Here, we sampled finches from two distinct populations across the MG invasion gradient: Virginia (VA) and Arizona (AZ). MG has been endemic in the VA population for at least twenty years longer than the Tempe, AZ population sampled for this study (Ley et al. 1996; Henschen et al. 2023), although MG was first detected in southern AZ in 2011 (Staley et al. 2018). Therefore, the two study populations will be referred to as MG-endemic and MG-naive, respectively. Specifically, inoculated finches from MG-endemic populations have shown higher tolerance (Adelman et al. 2013; Bonneaud et al. 2019; Henschen et al. 2023), quicker recovery (Bonneaud et al. 2019), and in some cases, lower MG burden (i.e., higher resistance; Bonneaud et al. 2011, but see Adelman et al. 2013; Henschen et al. 2023) compared to finches from MG-naive populations. This suggests a history of stronger MG-mediated selection in MG-endemic populations. If selection has favored robust immune memory over an effective initial response, we would predict a negative relationship between initial and second responses, as in the Protective Infection Hypothesis (Fig. 1). In this scenario, individuals from the MG-endemic population would show a stronger negative correlation between initial and second infection responses than individuals from the MG-naive population.

Host sex is another potential source of variation in the relationship between initial and second inoculation responses. Male vertebrates are generally predicted to have higher susceptibility and lower resistance to initial pathogen exposure than females (Klein and Flanagan 2016, but see Valdebenito et al. 2021); however, the role of host sex in predicting responses to multiple pathogen exposures is less clear. In the house finch-MG system, mortality was higher in adult male finches compared to adult females following initial MG emergence (Nolan et al. 1998), which could have resulted from sex differences in immune responses to MG or from sex-biased costs of similar immune responses. Several studies have shown higher pathology, pathogen load, and infection duration in MG-inoculated male finches, compared to inoculated females (Love et al. 2016; Moyers et al. 2018; Sauer et al. 2024). Conversely, other studies noted a female bias in pathology (Altizer et al. 2004; Leon and Hawley 2017), or no sex differences in MG infection responses at all (Sydenstricker et al. 2005; Thomason et al. 2017; Vinkler et al. 2018; Weitzman et al. 2021). Sex differences in host responses to MG therefore remain unclear; however, such differences could occur under either the Protective Infection Hypothesis or the Consistent Host Hypothesis, and our work here will help discern whether these hypotheses help clarify sex differences in MG responses.

We tested these predictions using an experimental approach in wild-caught finches of both sexes, captured from two populations that vary in MG endemism. All finches were given a standard priming dose (“initial inoculation”); after evaluating responses (i.e., susceptibility, resistance, and tolerance) to this initial exposure, we allowed finches to recover and then inoculated each finch a second time with one of four possible doses. Varying second doses were used to fulfill goals of earlier studies focused on heterogeneity in susceptibility to infection (see Methods); however, this approach adds ecologically relevant variation to our study, as MG loads encountered by wild finches at fomites are expected to vary widely (Adelman et al. 2013). Moreover, it ensures variation in observed pathogen load during secondary infection, which can prove highly valuable in estimating levels of tolerance (Råberg et al. 2009, Råberg et al. 2014, Henschen et al. 2023). We used a model selection approach to evaluate the extent to which initial inoculation responses, host population of origin, sex, and second inoculation dose predicted susceptibility, resistance, and tolerance to a second inoculation at the individual level.

## Methods and materials

### Experimental inoculations and data collection

We captured wild first-year house finches in Tempe, AZ and Blacksburg, VA during 2021 and 2022; see Supplement for full capture and care methods. During both years, birds were inspected for clinical signs of MG infection (see eye scoring description below) on days 3, 7, and 14 after arriving in captivity. After two to three weeks in captivity, we collected blood samples to test for the presence of anti-MG antibodies, using a commercially available kit (99–09,298, IDEXX, Westbrook, Maine) and previously published methods (Henschen et al. 2025). Only animals that showed no clinical signs before the experiments and no evidence of anti-MG antibodies (<0.061 optical density at 630 nm) were included in the study to help reduce the confounding effects of active or recent infection. This seropositivity threshold was chosen as a conservative estimate based on the absorbance of negative controls (see Supplement for further details). While finches typically recover from initial MG infection within 5–8 weeks (Grodio et al. 2012) and some develop seronegative IgY titers by 41 days following initial exposure (Garrett-Larsen et al. 2026), the combination of hatch-year age at capture and serostatus used here minimizes the possibility that finches included in the study experienced any recent MG infection.

**Fig. 2:**
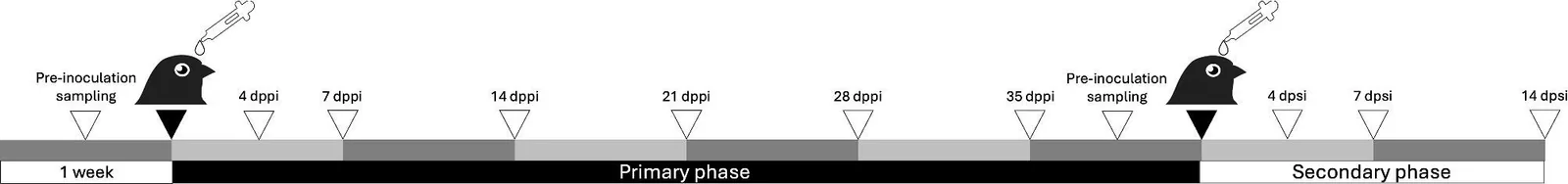
Experimental inoculation and sampling timeline of house finches (*Haemorhous mexicanus*) inoculated with *Mycoplasma gallisepticum* (‘MG’). White triangles indicate sampling dates and black triangles indicate inoculation dates (dpii = days post-initial inoculation, dpsi = days post-second inoculation). All finches were inoculated with an initial dose of 750 CCU/mL (color-changing units) of MG and one of four second doses (30,100, 300, or 7,000). Sampling included visual scoring of eye pathology and/or swabs for assessing MG load. See Methods and Materials for additional details.

The experiment included two inoculations with *Mycoplasma gallisepticum* strain VA1994 (NCSU ADRL 7994–1), which was isolated soon after the emergence of MG in wild finches (Ley et al. 1996). Using methods described previously (Hawley et al. 2024), all finches were inoculated with an initial dose of 750 color changing units (CCU)/mL of MG and, following recovery, with one of four doses (30,100, 300, or 7,000 CCU/mL) during the second inoculation. The data analyzed here from individuals in the MG-endemic population were also used in earlier studies focused on quantifying the impact of prior MG exposure on heterogeneity in susceptibility and disease responses (Hawley et al. 2024; Garrett-Larsen et al. 2026). However, those projects did not include the MG-naive population and focused solely on quantifying population-level variation in antibody responses and susceptibility to infection, rather than the individual-level patterns considered here. See Fig. 2 for a general experimental timeline and Table S1 for sample sizes by second MG dose, host population, and host sex.

Due to the constraints of housing wild-caught birds, the studies analyzed here occurred over two years and two institutions, all using identical methods. In 2021, finches from AZ and VA were inoculated at Virigina Tech, and in 2022, finches from AZ were inoculated at the University of Memphis; however, past work found no effect of institution location or sampling year on pathogen load or pathology (Henschen et al. 2023). During both years (2021 and 2022), the second inoculation took place 42 days after the initial inoculation, by which time most birds had recovered, no longer showing eye pathology or having a detectable MG load; those that did not recover were removed from analysis (see “Statistical Analysis” section). We used a visual scoring system to assess conjunctival pathology (“eye scores”; see timeline below) (Sydenstricker et al. 2005). Briefly, each eye was assessed on a scale from 0 (no visible pathology) to 3 (eye nearly swollen shut), and scores from both eyes were summed. We then collected conjunctival swabs to quantify MG load. The conjunctiva of each eye was swabbed using a sterile cotton swab wetted with sterile tryptose phosphate broth (TPB) for 5 s and then wrung out in a tube of sterile TPB (300 µL). Samples were stored at −20°C until DNA extraction. MG genomic DNA was extracted from each broth sample by using Qiagen DNeasy 96 Blood and Tissue Kits (Qiagen, Valencia, CA) and methods described previously (Hawley et al. 2013). We quantified MG load from extracted DNA by using a qPCR assay with primers targeting MG’s *mgc2* gene (Grodio et al. 2008) and methods as described previously (Hawley et al. 2013). All load values are transformed by log10(load + 1) unless otherwise noted.

We collected conjunctival eye swabs 7 days prior to initial inoculation, and on days 7, 40 or 41, 46, 49, and 56 (and on days 14 and 63 in 2021 only) following initial inoculation. Eye scores were collected 0, 7, 14, 21, 28, 35, 40 or 41, 46, and 56 days (and on day 49 in 2021 only) post-initial inoculation. These sampling dates were chosen based on an expected peak in MG pathology and load at seven days following inoculation (Adelman, Kirkpatrick, et al. 2013), with most birds recovered by 41 days following inoculation (Hawley et al. 2024).

### Quantifying inoculation responses

Susceptibility to both initial and second inoculation were binomial variables reflecting whether an individual was successfully infected following MG inoculation. Finches with an eye score >0 on 7, 14, 21, 28, 35, and/or 41 dpii (days post initial inoculation), and/or a pathogen load >15 copies on any of these dates, were considered successfully infected following initial inoculation. Similarly, finches with an eye score >0 on 4, 7, 14, and/or 21 dpsi (days post second inoculation), and/or a load >15 copies on any of these dates, were considered infected following the second inoculation.

Because resistance refers to the ability of an individual to control pathogen replication, we used qPCR-derived pathogen loads as a proxy for resistance. Thus, individuals with relatively lower pathogen loads were interpreted as more resistant than those with higher pathogen loads. Specifically, we quantified resistance as MG loads 7 days following initial and second inoculations (“initial load” and “second load,” respectively). Seven days post-inoculation was chosen because finches sampled in 2022 were missing load data from 14 dpii, and we wished to standardize the number of days post-inoculation for assessing initial and secondary resistance.

Tolerance is generally quantified by regressing a metric of fitness over pathogen load (Råberg et al. 2007). Here, we used eye pathology (i.e., eye score) as a proxy of survival fitness, as eye scores are associated with a reduced probability of survival in wild finches (Adelman et al. 2013). Thus, more tolerant individuals have lower eye scores per unit pathogen load. We quantified initial tolerance for each finch by extracting negative residuals of a binomial generalized linear model (GLM, *stats* package [R Core Team 2024]) of eye score on pathogen load using data from all birds successfully infected during the initial inoculation. Using the same approach, we quantified tolerance to the second inoculation using data from finches successfully infected during the second inoculation. We used negative residuals for interpretability, so that positive values indicate higher tolerance. Because combined eye scores are a categorical variable between 0 and 6, we divided the maximum eye score recorded over the first two weeks following inoculation by 6, to give a variable between 0 and 1, following the approach of (Henschen et al. 2023) for estimating MG tolerance in house finches. Our binomial GLMs for generating tolerance values predicted the maximum eye score recorded over the first two weeks following inoculation (divided by 6), using MG loads recorded 7 days post inoculation as the predictor variable (e.g., 7 days post initial inoculation loads to calculate initial tolerance, and 7 days post second inoculation loads to calculate second-inoculation tolerance). Note that hereafter, “maximum eye score” refers to the maximum eye score recorded over the two weeks immediately following a particular inoculation. For the initial inoculation, this is the maximum eye score between days 7 and 14 post-inoculation, and for the second inoculation, this is the maximum eye score between days 3, 7, and 14 post-inoculation; note that 7 dpsi eye scores were only available for 2021.

### Statistical analysis

We investigated factors predicting house finch susceptibility, tolerance, and resistance to the second inoculation with MG through a model selection approach using Akaike Information Criterion corrected for small sample sizes (AICc; (Burnham and Anderson 2004)). Finches that showed signs of infection immediately before the second inoculation (eye score >0 and/or MG load >15 copies on 41 or 42 dpii) were excluded from analyses of second inoculation responses (n = 7) to avoid possible confounding effects of an active infection on host responses to the second inoculation. Our threshold of 15 copies distinguishes between the maximum load observed in finches with an eye score of zero (14 copies) and the minimum load observed in finches with eye scores >0 (19 copies). We also removed data from finches with incomplete records (n = 4). All analyses were carried out in R version 2024.04.2 + 764 (R Core Team 2024).

For each model comparison, we investigated which factor or combination of factors best explained variation in second inoculation responses by building candidate models which included one or more of the following predictors: host population, host sex, and responses to the first (i.e., initial) MG inoculation, and second MG dose. Responses to initial inoculation included initial susceptibility (i.e., infected or uninfected during the first MG challenge), initial MG load, maximum initial eye score, and initial tolerance residuals (successfully infected finches only). Initial eye scores and resistance were not included in the same model to avoid collinearity. Note that dose was not included in candidate models for initial inoculation (see Supplement) because all finches received the same dose in initial inoculations. To avoid overfitting, all models included a maximum of one predictor per ten observations; interactive terms were included where sample sizes were thus large enough to avoid overfitting. We compared candidate models by AICc by using the “aictab” function in the *bbmle* package in R (Bolker et al. 2023). We assessed residuals of GLMs using the *DHARMa* package (Hartig et al. 2024) and inspected plotted residuals of linear models (*stats* package [R Core Team 2024]) visually; we also assessed the fit of multivariate models by ANOVA. We selected the top model for each analysis by excluding lower ranking models (> 2 ΔAICc) that were nested within higher-ranking models, models with simpler and higher-weighted variants (Richards et al. 2011), as well as any models that failed the goodness of fit checks. In most cases, this yielded no more than one top model per analysis, for which estimates are reported in the Results section. One analysis yielded three top models (see Table S5), and the top model by weight is reported in the Results.

### Statistical analysis: susceptibility to second inoculation

We first investigated factor(s) predicting susceptibility to the second MG inoculation (i.e., “successfully infected” or “not successfully infected”) in all finches (n = 84), using generalized linear models (GLMs) with a binomial error distribution. Because inoculum dose is strongly predictive of successful MG infection, all candidate models included second inoculation dose. Using a similar approach, we then investigated second inoculation susceptibility in the subset of finches that were susceptible to the first inoculation (n = 37), which allowed us to include initial tolerance as a potential predictor.

### Statistical analysis: resistance to second inoculation

Next, we investigated factor(s) predicting resistance in finches successfully infected (e.g., “susceptible”) in the second MG inoculation (n = 22), using linear models with a Gaussian error distribution. These analyses were restricted to finches that were successfully infected (e.g., “susceptible”) in the second inoculation. Within the MG-endemic population, relatively few birds (n = 2 of 9) that were successfully infected from the first inoculation became reinfected after the second inoculation, reducing our ability to investigate consistency in resistance within this group. Therefore, we ran a second analysis in finches from the MG-naive population only (n = 13 finches that were successfully infected from first inoculation and became reinfected). Candidate models included the same predictors as used for the earlier resistance analysis, apart from the exclusion of host population.

### Statistical analysis: tolerance of second inoculation

Finches that do not develop infection have no detectable eye pathology or MG load; therefore, these individuals would have similar tolerance residuals (i.e., very small) to successfully infected finches that had relatively low eye pathology scores. To avoid conflating these two scenarios, which likely reflect different physiological dynamics, we investigated predictors of second-inoculation tolerance values (calculations described above) only in animals who developed infection following second inoculation (n = 22), using linear models with a Gaussian error distribution. Candidate models included the same predictors as used for the resistance analysis. For the same reasons as outlined for analyses of resistance, we conducted a second analysis restricted to finches from the MG-naive population (n = 13). Similarly, candidate models included the same predictors listed above, apart from host population. Full candidate model details for each model comparison, as well as results of models < 2 ΔAICc for each analysis are shown in Lists A-F and Tables S2-S7, respectively, in the Supplement. Analyses for finch responses to initial inoculation (i.e., initial susceptibility, resistance, and tolerance) are available in the Supplement (“Responses to Initial Inoculation,” Figs S1-S3, Lists G-J, and Tables S8–11).

## Results

### Susceptibility to the second inoculation

**Fig. 3:**
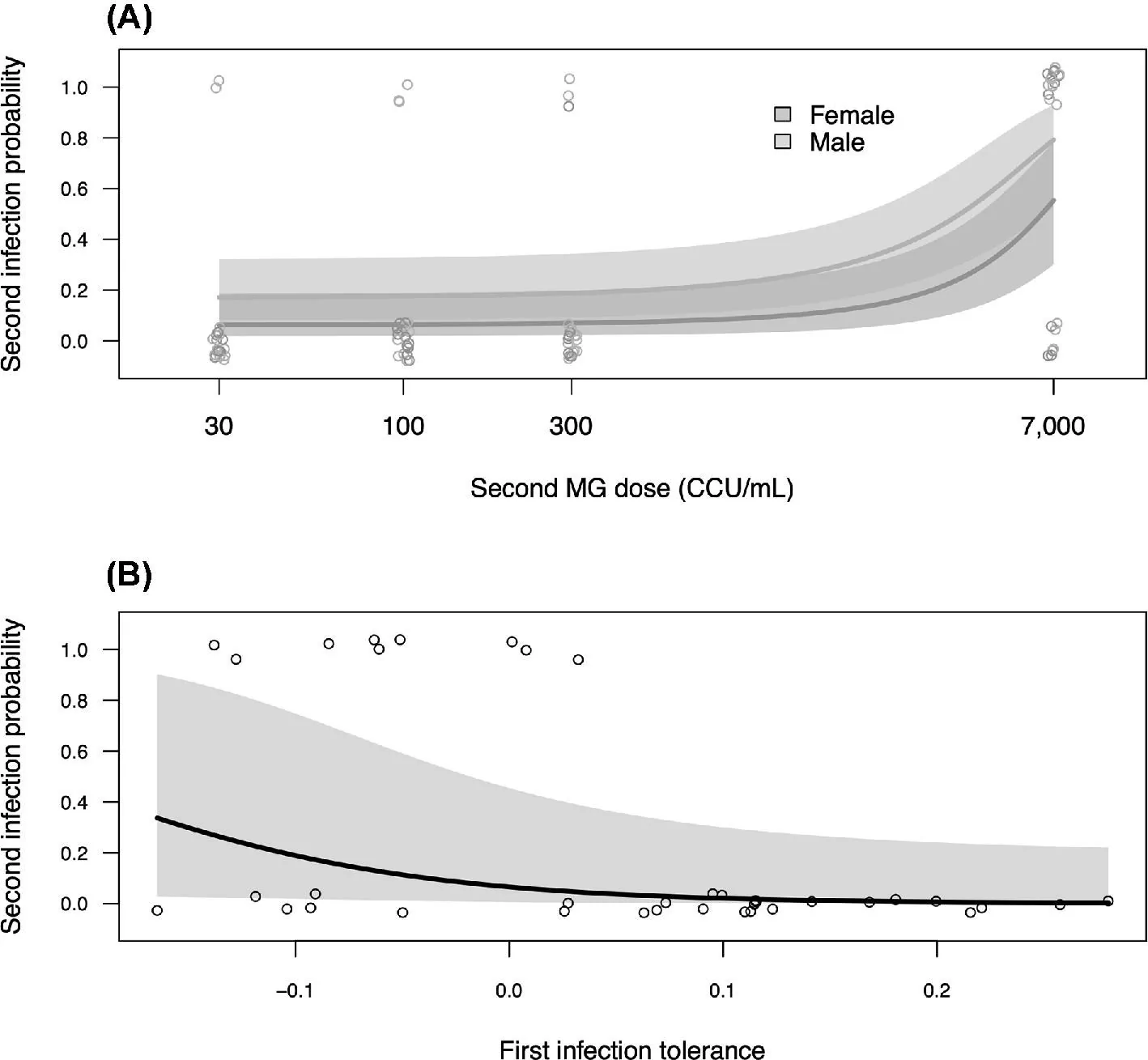
Model predictions (lines) for a generalized linear model predicting the probability of infection following a second inoculation of *Mycoplasma gallisepticum* (MG) in house finches (*Haemorhous mexicanus*; n = 84). Panel (A) infection probability at all second MG doses is higher for male (shown in yellow) than female finches (blue). Panel (B) among finches successfully infected during their first MG exposure (n = 37), those with higher tolerance during the first infection had a lower probability of infection following a second MG exposure. In both plots, ribbons represent 95% confidence intervals for each model and individual points, representing data for individual finches, are jittered for easier viewing.

Among all finches (n = 84), the best supported model predicting susceptibility to the second inoculation included second inoculation dose and host sex. The fitted coefficients reflect a positive correlation between susceptibility and the dose a finch received at second inoculation (Est = 4.18^−4^ ± 9.58^−5^ SE; Table S2). The model also suggests higher susceptibility in male finches compared to females (Fig. 3A; Est = 1.12 ± 0.65 SE). Initial inoculation susceptibility was not present in any models with < 2 ΔAICc, suggesting that it was not an important predictor of individual susceptibility to the second inoculation (Table S2; see List A in Supplement for a full list of candidate models and Table S1 for sample sizes by second MG dose, host sex, and host population). Model comparisons for initial susceptibility are available in the Supplement.

Among finches successfully infected following initial inoculation (n = 37), the best-supported model included second inoculation dose, host sex, population of origin, and initial tolerance. Fitted coefficients suggest second-inoculation susceptibility increased with second MG dose (Est = 5.11^−4^ ± 2.50^−4^ SE), was higher for male finches compared to females (Est = 3.61 ± 1.89), was lower for those with higher initial tolerance (Est = −12.04 ± 6.55), and was also lower in MG-endemic finches compared to MG-naive finches (Est = −2.62 ± 1.39; see Table S3 and Fig. 3B). See Table 2 for a summary of main results and List B in the Supplement for a full list of candidate models.

**Table 2:** Summary table of responses of house finches (*Haemorhous mexicanus*) to a second experimental inoculation with *Mycoplasma gallisepticum* bacteria (“MG”). Responses (black column) are given in terms of susceptibility (S), resistance (R), and tolerance (T); see Table 1 for full definitions of each term. Potential predictors of each response type are listed in the top grey row. Those selected by model comparison as important predictors of each response are listed; blank light grey spaces indicate the factor was not selected as an important predictor for a given response. Example interpretation, “Tolerance to a second inoculation is higher with higher second MG doses in finches from Arizona.” Results restricted to finches that were susceptible to the initial inoculation are indicated with a diamond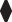. Results restricted to finches from the MG-naive population (AZ) are indicated with an asterisk*.

Response to second inoculation	Second MG dose	Host sex	Host population	Initial susceptibility	Initial resistance	Initial tolerance	Initial pathology
**Susceptibility** (Probability of infection)	Higher susceptibility with higher second dose	Higher susceptibility in males than females	Lower susceptibility in finches from MG-endemic population (VA)^ 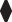 ^			Lower susceptibility with higher initial tolerance^ 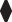 ^	
**Resistance** (Higher with lower MG load)	Lower resistance with higher second dose			Higher resistance in initial susceptible than in initial unsusceptible	Higher resistance with lower initial resistance*		
**Tolerance** (Higher with less pathology per unit pathogen load)	Higher tolerance with higher second dose*			Higher tolerance in initial susceptible than in initial unsusceptible*			

### Resistance to the second inoculation

**Fig. 4:**
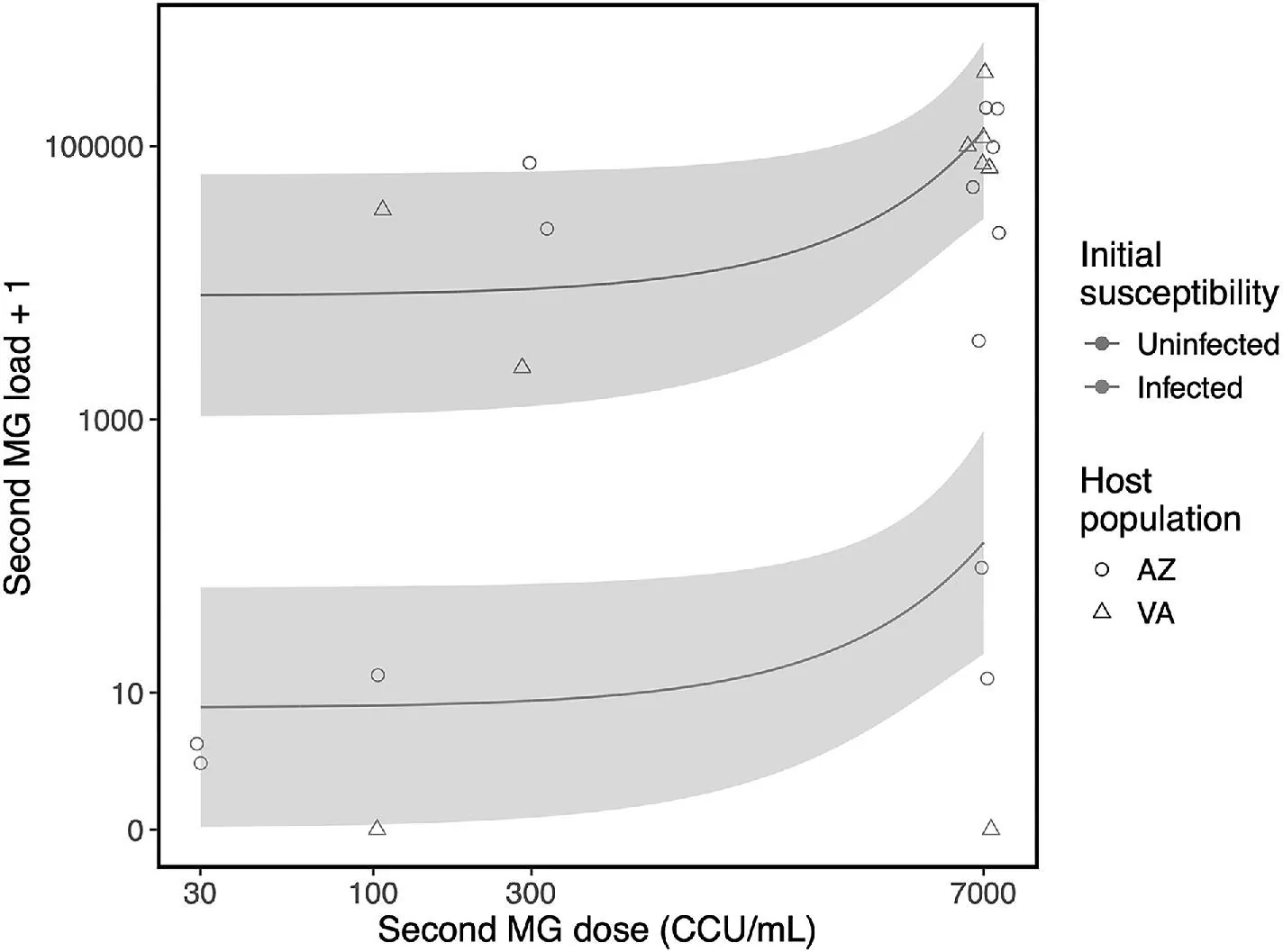
In house finches (*Haemorhous mexicanus*) inoculated sequentially with *Mycoplasma gallisepticum* (MG) bacteria, resistance (measured as MG load, log10-transformed on the y-axis) following the second inoculation is relatively higher in finches that became infected and developed relatively higher loads following the initial inoculation, and also varies with MG dose (x-axis). Shown are predictions and 95% confidence intervals from a linear model of resistance with second MG inoculum dose and initial susceptibility status as predictors (i.e., successfully infected following initial inoculation, in orange, or not successfully infected, in purple). Points represent data from individual finches (n = 22) and are jittered for ease of viewing. Although host population is not included in the model, points are shaped by host population (AZ in circles, VA in triangles). Note that the x-axis is log10 transformed for easier viewing, but doses are not transformed in the model.

Among finches that were successfully infected following the second inoculation (n = 22), the best-supported model included second inoculation dose and initial susceptibility as predictors of second inoculation resistance. Fitted coefficients indicate a positive correlation between second inoculation MG loads (i.e., lower resistance) and second inoculation dose (Est = 1.73^−4^ ± 6.99^−5^ SE). In addition, the top model suggests that finches successfully infected following initial inoculation had lower second loads (i.e., higher resistance) compared to finches that were not successfully infected following initial exposure (Est = -3.02 ± 0.47 SE); see Fig. 4 and Table S4. See List C in the Supplement for a full list of candidate models. Similar analyses for initial resistance are available in the Supplement.

Among finches from the MG-naive population (n = 13), the best-supported model included only initial susceptibility as a predictor of second inoculation resistance. Fitted coefficients from this model indicate that finches which were successfully infected following initial inoculation had lower secondary loads (i.e., higher resistance) compared to finches that were not successfully infected following initial exposure (Est = −2.75 ± 0.74 SE). Two other models with < 2 ΔAICc that showed higher initial loads and eye scores, respectively, were associated with lower second loads, likely because of their close relationship with initial infection status; see Table S5. Note that these results reflect a top model set of three univariate models and that the modest sample size prohibited including a full model in the model comparison; see Table S5.

### Tolerance of the second inoculation

Among finches that became successfully infected following the second inoculation (n = 22), none of our predictors (sex, host population, responses to initial infection, and second dose) improved predictions of second-inoculation tolerance compared to a null model. See Table S6 for details. Similar analyses for initial tolerance are available in the Supplement.

**Fig. 5:**
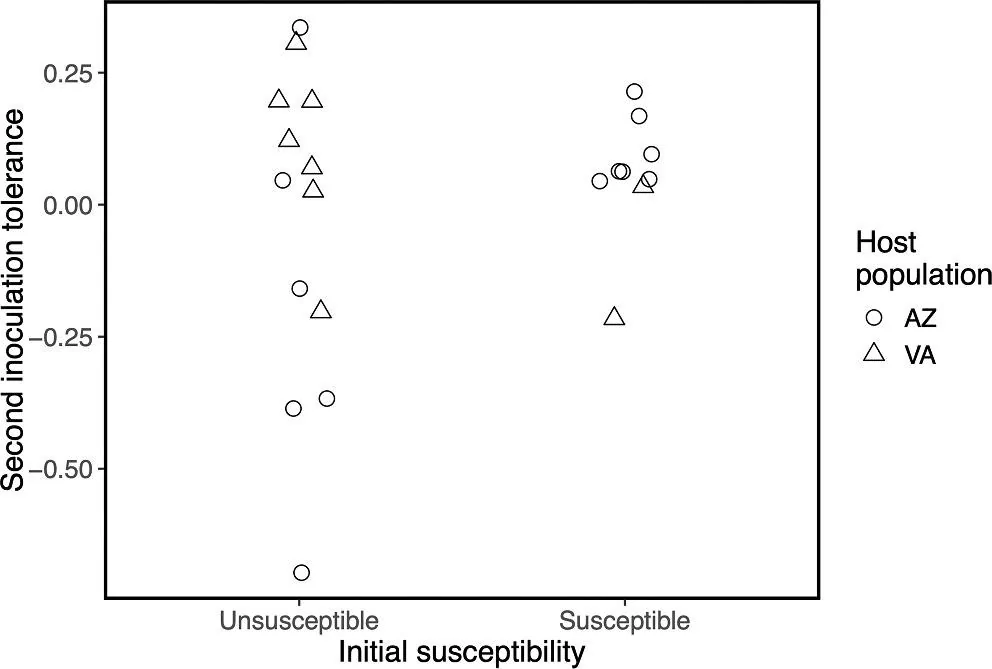
In house finches (*Haemorhous mexicanus*) from a host population (AZ) naïve to *Mycoplasma gallisepticum* (MG) at the time of the study, tolerance following a second MG inoculation was higher in finches that were susceptible to initial inoculation (n = 7), compared to those not susceptible to initial inoculation (n = 6). Data are also shown for finches from a population where MG has been endemic (VA) for several decades (triangles, n = 9), but these data were not included in the analysis of second inoculation tolerance due to low numbers of VA finches that were susceptible to initial inoculation. Tolerance is quantified as the residuals from a GLM predicting eye pathology from MG load; see Methods and Materials for additional details. Points represent tolerance values for individual finches.

However, among MG-naive finches (n = 13), the best-supported model included initial susceptibility and second inoculation dose as predictors of second inoculation tolerance. Fitted estimates suggest that finches which were successfully infected following initial inoculation had higher second-inoculation tolerance than those that were not successfully infected following initial inoculation (Est = 0.33 ± 0.12). There was also a positive relationship between second MG dose and tolerance (Est = 3.86^−5^ ± 1.82^−5^); see Fig. 5. However, it should be noted that the null model was also included in the top model set (ΔAIC = 1.74, weight = 0.12), and therefore the results of the top model should be interpreted cautiously; see Table S7.

## Discussion

Here we used an experimental approach in wild-caught house finches to investigate whether host responses to an initial inoculation with *Mycoplasma gallisepticum* (“MG”) were predictive of responses to a second inoculation, and whether this relationship varied by host population of origin and/or sex. Specifically, we tested the hypotheses that individual host’s responses to a second inoculation with the same pathogen were (a) negatively associated with initial responses (“Protective Infection Hypothesis”), or (b) relatively consistent with their responses to initial inoculation (“Consistent Host Hypothesis”). If the Protective Infection Hypothesis were true, we would expect finches that developed infection and became the sickest in response to initial exposure would have a relatively lower probability of infection and/or would be less sick (i.e., show higher resistance and/or tolerance) following the second inoculation (see Fig. 1). Conversely, under the Consistent Host Hypothesis, we would predict that individuals who became infected and were the sickest (i.e., low tolerance and/or resistance) following initial inoculation would also develop infection and become the sickest after the second inoculation.

We found moderate support for the Protective Infection Hypothesis. Our best-supported model of second inoculation resistance suggested that finches successfully infected by the first MG inoculation had lower second MG loads than those that were not, suggesting that susceptibility to initial inoculation increased second-inoculation resistance. Relatively few finches from the MG-endemic population (i.e., those from VA) that were susceptible to their first inoculation contracted a second infection, reducing our ability to compare the relationship between initial and second infection resistance in this group. However, among finches from the MG-naive population (i.e., those from AZ), the best supported model suggested a negative relationship between initial and second loads, indicating that AZ finches with the lowest initial resistance showed the highest resistance to the second inoculation. Similarly, the best-supported model of second inoculation tolerance in AZ finches suggested higher second-inoculation tolerance in finches susceptible to initial inoculation. Together, these results support the Protective Infection Hypothesis prediction that finches with a more severe initial infection would develop stronger protection, for example through stronger immune memory, and thus experience less severe second infections. Similarly, humans infected with the novel SARS-Cov-2 virus show a positive relationship between disease severity and the production of neutralizing antibodies (Garcia-Beltran et al. 2021). Additional work investigating the Protective Infection Hypothesis is warranted and would benefit from including additional axes of infection response, such as time to recovery and area under the curve of pathogen load over the course of infection.

In comparison, we found relatively less support for the Consistent Host Hypothesis. The only supporting evidence for this hypothesis was that the best-supported model for second inoculation susceptibility included initial tolerance as a predictor; estimated coefficients suggested higher second inoculation susceptibility in finches with lower initial tolerance. However, we did not observe individual consistency in susceptibility: the numbers of individuals that were consistently susceptible (double-infection group) or unsusceptible to MG inoculation (zero-infection group) were consistent with a null model in which probability of second infection was independent of probability of first infection, and thus initial susceptibility was not predictive of second susceptibility. Rather, the existence of the zero-infection and double-infection groups is better explained by variation in second MG doses, as the probability of infection following the second inoculation increased with higher second MG doses, which is an expected consequence of such increased pathogen pressure (Hawley et al. 2024). Similarly, we found no support for individual consistency in resistance or tolerance, leading us to the conclusion that the Protective Infection Hypothesis is better supported in this system than the Consistent Host Hypothesis. This is in contrast to recent work that has demonstrated individual consistency in immune profiles of wild mammalian hosts (Arriero et al. 2017; Corripio-Miyar et al. 2025). For example, in Soay sheep (*Ovis aries*), interleukin-4 and interferon-gamma showed longitudinal consistency and were positively associated with resistance to gastrointestinal helminths and coccidia, respectively (Corripio-Miyar et al. 2025). Therefore, the probability of individual consistency in host responses to parasitism might vary by host taxon or host-pathogen dynamics (i.e., sterilizing immunity vs. chronic infection). Additional work is needed to address these possibilities, as well as the role of infection timing; for example, recent work suggests protective effects of early-life infection on host fitness, which could reflect long-term effects of the Protective Infection Hypothesis (Knutie et al. 2025).

One might predict that developing a relatively severe initial infection to avoid a severe second infection (i.e., Protective Infection Hypothesis) would be selectively favored when the risk of repeated exposure is high (Cressler and Adelman 2024). However, finches with relatively higher tolerance to initial infection showed reduced second-inoculation susceptibility, which would be consistent with some combination of both hypotheses, wherein some individuals may be consistently better protected than others, while also gaining additional protection from first exposure. Further, this could be directly in line with the Consistent Host Hypothesis as outlined in Fig. 1 if the observed lack of susceptibility was actually a manifestation of very rapid infection clearance. Our results suggest that there are multiple scenarios in which individuals may avoid severe future infections, both of which reflect a multi-step selection process, requiring that individuals survive a successful initial infection. This may help explain the persistence of individual variation in immunopathology within host populations where MG has become endemic (Cressler and Adelman 2024). Additional work is needed to determine whether these patterns are general across finch populations and MG strains, as well as other host-pathogen systems.

It is also important to note that relatively few finches were susceptible to both inoculations (n = 9), reducing our power to detect individual consistency in tolerance. Therefore, it remains unclear whether tolerance is a repeatable trait at the individual level and further work is needed to address this question. In addition, we only quantified tolerance in successfully infected finches, rather than assigning high tolerance values to uninfected finches. We adopted this approach because distinct processes could yield similar tolerance values when uninfected individuals are included in the analysis. For example, because tolerance scores are quantified as a residual of a model predicting eye score from load, an uninfected individual with load and eye score of zero would have a similar score as any infected individual with load and pathology scores along the reaction norm. Therefore, to distinguish these processes, we recommend that researchers analyze infected and uninfected groups separately in studies that address individual variation in host tolerance. In addition, we note that by removing individuals that had not recovered from the initial infection from our analyses, we may have removed some of the least tolerant individuals; future work might incorporate a longer recovery period to avoid this.

### Responses to second MG inoculation by host population and sex

Because prior work in the house finch-MG system showed higher MG tolerance and resistance in finches from MG-endemic versus MG-naive populations (Adelman et al. 2013; Bonneaud et al. 2019; Henschen et al. 2023), we predicted that the Protective Infection Hypothesis was more likely to be supported in the MG-endemic population (VA), compared to the MG-naive population (AZ). Conversely, if the Consistent Host Hypothesis were supported, we predicted that finches from the MG-endemic population would show higher resistance and tolerance following a second inoculation, compared to finches from the MG-naive population.

Neither of these hypotheses were directly supported, as population was not selected as an important predictor of tolerance or resistance to a second MG inoculation. It should be noted that relatively few MG-endemic finches were susceptible to the second inoculation, reducing our ability to detect population differences in second-inoculation tolerance. That said, the probability of a second infection was higher in finches with relatively lower initial tolerance, and the MG-naive population had relatively lower initial tolerance than the MG-endemic population. Indeed, in an analysis restricted to finches susceptible to the first inoculation, the best-supported model suggested that finches from the MG-naive population had a higher probability of infection following the second inoculation, relative to MG-endemic finches. Together, these results may hint at a subtle signal of selection: that the MG-endemic population contains a greater proportion of finches that show relatively high tolerance of an initial MG inoculation and reduced susceptibility to a second MG inoculation. Indeed, reduced susceptibility to subsequent infection may be an underappreciated benefit of tolerance in other wild vertebrate populations where tolerance to novel pathogens has evolved.

Within populations, host responses may also vary by sex. Although we found no effect of sex on initial susceptibility (see Supplement), the best-supported model for second inoculation susceptibility included sex as a predictor and the estimated effect size was larger and more precise when considering finches successfully infected during the first inoculation. This may indicate that females experience stronger protection from an initial infection under the Protective Infection Hypothesis. Stronger protection could be mediated by a female bias in immune memory, despite prior work suggesting a male bias in MG-specific antibody production in MG-inoculated songbirds (Gates et al. 2021; Sauer et al. 2024). Future work should address possible sex differences in antibody persistence and localization.

A male bias in second-inoculation susceptibility could have important implications for transmission. Male-driven pathogen transmission is well demonstrated in mammals (Ferrari et al. 2003; Grear et al. 2009; VanderWaal et al. 2013; Leu et al. 2020), although this process has received relatively little attention in avian systems beyond sexually transmitted infections (Sheldon 1993; Kulkarni and Heeb 2007). Previous experimental work demonstrated faster MG transmission among male songbirds than females after a single MG inoculation (Adelman et al. 2015; Sauer et al. 2024). Male finches also prefer to feed near MG-infected, rather than healthy, conspecifics, while females show no preference (Bouwman and Hawley 2010); this behavior creates an opportunity for male-biased transmission at bird feeders, where MG transmission is known to occur (Dhondt et al. 2007; Adelman et al. 2015; Moyers et al. 2018). Together, these studies and our results suggest that males could disproportionately drive MG transmission in wild finches, with a potential for complex interactions with exposure history.

Beyond susceptibility, we found no sex differences in second-inoculation responses (i.e., tolerance or resistance). Similarly, we found no sex differences in responses to initial inoculation (see Supplement); together, these results argue against the Consistent Host Hypothesis in terms of sex. Interestingly, we noted lower initial loads in MG-endemic females compared to MG-naive females (see additional analysis in the Supplement), indicating relatively higher initial resistance in MG-endemic females. This sex by population interaction in individuals with no prior exposure, who have no acquired immunity, could reflect sex-specific selective pressure of MG on finch innate immunity. Indeed, female-biased resistance is commonly documented in vertebrates (Klein and Flanagan 2016), although the generality of this pattern in birds has been challenged (Kelly et al. 2018; Valdebenito et al. 2021, 2024). More work is needed to determine whether there are sex-specific physiological benefits of high initial resistance to MG in MG-endemic populations. Given the trend of higher second-inoculation susceptibility of males (Fig. 3A), it is possible that initial resistance is more adaptive in females than males; because females are less likely to experience second infections, limiting pathogen replication during the initial infection may be advantageous. If this hypothesis is borne out, the finch-MG system would comprise a rare opportunity to study sex-specific selective pressures of emerging pathogens on host immune function in wild vertebrates.

### Broader implications for epidemiology and host-pathogen coevolution

Our findings have important implications for transmission and host adaptation to emerging pathogens. We found greater support for the Protective Infection Hypothesis than the Consistent Host Hypothesis, which broadly suggests that individual hosts in this system are not consistent in their relative levels of response to repeated pathogen exposure. This indicates possible shifts in individual hosts’ contribution to pathogen transmission (i.e., competence). Relative to finches that were unsusceptible to an initial inoculation, finches that were successfully infected by initial inoculation had lower second MG loads (i.e., higher resistance) and, in the case of birds from the MG-naive population, less pathology per unit pathogen load (i.e., higher tolerance) following their second inoculation. These shifts could facilitate individual transitions from high-competence to low-competence, as the probability of MG transmission increases with pathogen load, regardless of prior exposure (Leon et al. 2025), and is negatively associated with pathology (Adelman, Carter, et al. 2013; Bonneaud et al. 2020; Sauer et al. 2024). Thus, finches susceptible to their first MG exposure may play a relatively minor role in MG transmission during subsequent exposures, perhaps even helping to mute transmission. For example, recent work shows reduced transmission by MG-inoculated finches with higher tolerance (as measured by severity of pathology (Ruden and Adelman 2021; Henschen et al. 2025)). Conversely, finches that are not susceptible to their first MG exposure may play a relatively larger role in MG transmission during subsequent exposures, as they showed relatively higher second-inoculation loads. Accounting for host role reversal across pathogen exposures may help improve the accuracy of epidemic models in this and other host-pathogen systems.

Our results also have implications for host adaptation to emerging pathogens. We suggest that finches face alternative strategies following an initial MG exposure: a) develop an active infection and reduce the probability and severity of a future infection or b) fully resist an initial infection and risk developing a severe future infection. The relative benefits of each strategy could vary by several factors, including the probability of future MG exposures, the availability of resources needed for reducing pathogen replication and repairing tissues damaged through immunopathology, competing physiological demands on available resources (e.g., reproduction, thermal regulation, coinfections, etc.), and the durability of the protective benefit of initial infection. Although MG prevalence peaks seasonally, it is present year-round in MG-endemic finch populations (Altizer et al. 2004); thus, we predict that the probability of repeated MG exposure is moderate to high for individual finches. Therefore, selection may favor individuals that are susceptible to initial MG exposure, compared to those that are not, because of the benefit of reduced infection severity following future pathogen exposure. Furthermore, the benefit of this strategy may vary by sex. Additional work in this system and others is needed to understand sex-specific costs and benefits of different responses to initial pathogen exposure, especially during different seasons, when hosts face variation in pathogen pressure, as well as competing demands on physiological resources (e.g., immune function and reproduction).

## Supplementary Material

obag050_Supplemental_Files

## Data Availability

Data and code are deposited on https://github.com/talbottkm/mg_response_pop_sex/.
