# Supplementary material for: Paying Upfront: Successful Initial Infections Protect Against Severe Future Infections
Source: Integr Org Biol. 2026 Aug 21;8(1):obag050. doi: 10.1093/iob/obag050 (PMC13583131; doi:10.1093/iob/obag050)
Supplement: Supplementary file 1 [file obag050_supplemental_files.zip › AltText_009.docx]

**Cover image:** A photograph of three house finches (small brown and red songbirds) perched on a bird feeder outdoors, eating sunflower seeds. One finch in the foreground has a swollen eye, typical of *Mycoplasma gallisepticum* infection.

**Figure 1**: A conceptual figure visualizing relationships between initial and second responses of house finches to *Mycoplasma gallisepticumi* infection. The x and y axes are the same, with ‘uninfected despite exposure’ near the 0,0 intercept, ‘high tolerance and/or resistance’ in the middle of the axes, and ‘low tolernace and/or resistance’ on the most distal point of the axes. The ‘Consistent Host Hypothesis’ predicts a positive slope between initial and second inoculation responses, while the ‘Protective Infection Hypothesis predicts a negative slope between the two responses.

**Figure 2**: A figure representing the experimental timeline. A central line is sectioned into nine blocks, with each block representing one week. An icon of a bird is placed after the first week, indicating the day of initial inoculation, and another bird icon is placed after the seventh week, indicating the day of the second inoculation. Small triangles are placed along the timeline to indicate days of sampling (see methods).

**Figure 3**: A two-paneled figure. The top figure depicts the predicted relationship between the probability of successful infection following the second inoculation on the y axis and second inoculation dose on the x axis. Two different colored lines distinguish predicted relationships by sex, with each line accompanied by a ribbon representing the 95% confidence interval. Individual data points are overlaid on the graph. The bottom figure depicts the predicted relationship between the probability of successful infection following the second inoculation on the y axis and initial inoculation tolerance values on the x axis. One line represents the predicted relationship for all birds successfully infected following the first inoculation and is accompanied by a ribbon representing the 95% confidence interval. Individual data points are overlaid on the graph.

**Figure 4**: A figure representing the relationship between *Mycoplasma gallisepticum* (MG) loads in individual finches following their second inoculation on the y axis and second inoculation MG dose on the x axis. Two different colored lines distinguish predicted relationships by sex, with each line accompanied by a ribbon representing the 95% confidence interval. Individual data points are overlaid on the graph. Points are further distinguished by shape, with finches from Virginia, an MG-endemic population, represented by triangles, and finches from Arizona, a relatively naïve population, represented by circles.

**Figure 5**: A scatterplot depicting the relationship between tolerance of a second *Mycoplasma gallisepticum* inoculation on the y axis and initial susceptibility on the x axis. Initial susceptibility is broken into two categories, finches that were unsusceptible to their first inoculation (on the left), and those susceptible to the first inoculation (on the right). Individual data points are shown; finches from Virginia, an MG-endemic population, re represented by triangles, and finches from Arizona, a relatively naïve population, represented by circles.
