# Supplementary material for: Paying Upfront: Successful Initial Infections Protect Against Severe Future Infections
Source: Integr Org Biol. 2026 Aug 21;8(1):obag050. doi: 10.1093/iob/obag050 (PMC13583131; doi:10.1093/iob/obag050)
Supplement: Supplementary file 1 [file obag050_supplemental_files.zip › FigureCaptions_009.docx]

**Figure 1.** Hypothesized relationships between initial and second inoculation responses in individual house finches (*Haemorhous mexicanus*) inoculated with *Mycoplasma gallisepticum* (MG) bacteria twice. Note that responses form a gradient of infection severity, not discrete response categories, and that the ‘uninfected’ group represents individuals that are exposed to MG but do not show evidence of infection (i.e., detectable load and/or eye score). Prediction line slopes are shallower than a perfect regression to reflect the effect of acquired immune responses to the initial inoculation on responses to the second inoculation (i.e., that all finches successfully infected following initial inoculation should gain some level of protection).

**Figure 2.** Experimental inoculation and sampling timeline of house finches (*Haemorhous mexicanus*) inoculated with *Mycoplasma gallisepticum* (‘MG’). White triangles indicate sampling dates and black triangles indicate inoculation dates (dpii = days post-initial inoculation, dpsi = days post-second inoculation). All finches were inoculated with an initial dose of 750 CCU/mL (color-changing units) of MG and one of four second doses (30, 100, 300, or 7,000). Sampling included visual scoring of eye pathology and/or swabs for assessing MG load. See Methods and Materials for additional details.

**Figure 3.** Model predictions (lines) for a generalized linear model predicting the probability of infection following a second inoculation of *Mycoplasma gallisepticum* (MG) in house finches (*Haemorhous mexicanus*; n = 84). Panel A) infection probability at all second MG doses is higher for male (shown in yellow) than female finches (blue). Panel B) among finches successfully infected during their first MG exposure (n = 37), those with higher tolerance during the first infection had a lower probability of infection following a second MG exposure. In both plots, ribbons represent 95% confidence intervals for each model and individual points, representing data for individual finches, are jittered for easier viewing.

**Figure 4.** In house finches (*Haemorhous mexicanus*) inoculated sequentially with *Mycoplasma gallisepticum* (MG) bacteria, resistance (measured as MG load, log10-transformed on the y-axis) following the second inoculation is relatively higher in finches that became infected and developed relatively higher loads following the initial inoculation, and also varies with MG dose (x-axis). Shown are predictions and 95% confidence intervals from a linear model of resistance with second MG inoculum dose and initial susceptibility status as predictors (i.e., successfully infected following initial inoculation, in orange, or not successfully infected, in purple). Points represent data from individual finches (n = 22) and are jittered for ease of viewing. Although host population is not included in the model, points are shaped by host population (AZ in circles, VA in triangles). Note that the x-axis is log10 transformed for easier viewing, but doses are not transformed in the model.

**Figure 5.** In house finches (*Haemorhous mexicanus*) from a host population (AZ) naïve to *Mycoplasma gallisepticum* (MG) at the time of the study, tolerance following a second MG inoculation was higher in finches that were susceptible to initial inoculation (n = 7), compared to those not susceptible to initial inoculation (n = 6). Data are also shown for finches from a population where MG has been endemic (VA) for several decades (triangles, n = 9), but these data were not included in the analysis of second inoculation tolerance due to low numbers of VA finches that were susceptible to initial inoculation. Tolerance is quantified as the residuals from a GLM predicting eye pathology from MG load; see Methods and Materials for additional details. Points represent tolerance values for individual finches.
