# Supplementary material for: Paying Upfront: Successful Initial Infections Protect Against Severe Future Infections
Source: Integr Org Biol. 2026 Aug 21;8(1):obag050. doi: 10.1093/iob/obag050 (PMC13583131; doi:10.1093/iob/obag050)
Supplement: Supplementary file 1 [file obag050_supplemental_files.zip › Supplement_009.docx]

# Paying upfront: successful initial infections protect against severe future infections

# Supplement

### Finch capture and care

From June - August 2021, juvenile house finches were captured in and near Blacksburg, Virginia (VA) and Tempe, Arizona USA (AZ) using baited feeder traps and mist nets. Finches showing signs of conjunctivitis were released at the site of capture; all others were fitted with a metal, uniquely numbered leg band for identification. Finches captured in VA were transported to Virginia Tech via automobile using approved transport methods; finches captured in AZ were transported to Arizona State University in similar fashion. Finches from AZ were held individually in cages (60 × 40 × 30 cm) in the Arizona State University vivarium for 2-8d, depending upon day of capture. These animals were transported via commercial aircraft to the University of Memphis in International Air Transport Association (IATA)‐approved pet carriers that had been modified to hold birds. Briefly, each carrier (67 x 46 x 52 cm) included five perches, absorbent material on the floor, and sufficient seed and fruit for finches to remain satiated and hydrated during the journey. Airline regulations require that no water be present in the cage, which is why we substituted fruit. Each carrier housed a maximum of 20 animals and the total time in transit was approximately 20 hours.

We also captured finches in Tempe, AZ in 2022, using the same procedures as above. However, because of airline cancellations, we transported finches to the University of Memphis via automobile in the same carriers as above, with water and seed bowls added to the carriers. This journey took approximately 22 hours and animals were inspected visually for any signs of distress at least every 4 hours. No animals died during transit in either year.

Upon arrival at the University of Memphis or Virginia Tech, animals were housed either individually or in pairs in cages (76 x 46 x 46 cm) with *ad libitum* access to water, grit, and food (a 20:80 blend of black oil sunflower seed:Roudybush Maintenance Nibbles; Roudybush, Inc., Woodland, CA). Rooms were maintained at a lighting regime of 12 hrs light: 12 hrs dark and constant temperature of approximately 22°C. Animals were treated prophylactically to minimize impacts of common parasites found in wild birds. To reduce risk of trichomoniasis, all birds received Cankerex (MedPet, Newport, WA, USA) in their only drinking water sources at 1 g/L for their first five days in captivity. Following this treatment, to minimize risk of coccidiosis, birds were provided with Endocox in their drinking water at 1.32g/L (L; 2.5% 99 toltrazuril, Jedd’s Bird Supplies, Anaheim, California, USA) for three consecutive days each week for four weeks. The other four days of each of these weeks, birds were provided with 1 g/L probiotics in their drinking water (Bene-Bac Plus bird and reptile supplement, Pet Ag, Inc., Hampshire, IL USA). After four weeks, Endocox was provided as above for three consecutive days every other week. Probiotics were provided twice weekly in doses as above.

### Assessing seropositivity in wild-captured finches

We selected 0.061 as our threshold for seropositivity based on the absorbance values for negative controls (included in the IDEXX assay). This threshold is conservative, as it is 1.76 SD away from the mean absorbance of negative controls, based on 338 individual wells from 109 plates measured on 79 separate dates across six years.

### Supplemental analyses: Responses to initial inoculation

We investigated factors predicting house finch susceptibility, tolerance, eye pathology, and resistance to initial inoculation with *Mycoplasma gallisepticum* (MG) by using an information theoretic model selection approach. This dataset is restricted to finches inoculated with an initial dose of 750 CCU of MG (n=116). This is a larger dataset than used for the main manuscript, as it includes some birds that were given control doses for the second inoculation for the purposes of a separate study (Hawley et al. 2024), and these individuals were excluded from central analyses. We used the same criteria as described in the methods of the main text to assess infection status, initial loads (i.e., resistance), initial tolerance, and maximum initial eye score.

For each analysis, we generated a list of candidate linear models (LMs *stats* package, R Core Team), generalized linear models (GLMs *stats* package, R Core Team), or ordinal regressions (*MASS* package, Ripley and Venables 2002) as appropriate and compared them by AICc by using the ‘aictab’ function (*bbmle* package; [(Bolker et al. 2023)](https://www.zotero.org/google-docs/?anDdly)). Predictors included host sex, population, and the interaction between host sex and population. Models <2 ΔAICc for each analysis are reported. In cases where the null has the highest weight, no other models are reported. In cases where there are multiple models <2 ΔAICc and the top model (i.e., highest weighted) is also the most parsimonious, only the top model is reported [(Richards 2008; Richards et al. 2011)](https://www.zotero.org/google-docs/?1coEmg). In cases where there are multiple models <2 ΔAICc, and lower weighted models are nested in the top model, only the top model is reported. For top models, we assessed residuals of GLMs by using the *DHARMa* package [(Hartig et al. 2024)](https://www.zotero.org/google-docs/?vUURY8) and visually inspecting residuals of LMs. During our analysis of initial resistance, visualization of the data indicated a pattern suggestive of a sex by population interaction. Therefore, we added a Kruskall-Wallis test to address this question, which was followed by a Dunn post-hoc test with a Benjamini-Hochberg correction for multiple tests. All analyses were carried out in R version 2024.04.2+764 (R Core Team 2024).

#### A) Model comparison: Susceptibility to initial inoculation

Among all finches (n=116), none of our predictors (sex, host population, and the interaction of sex and population) improved predictions of susceptibility to an initial MG inoculation, compared to a null model. See Table S8 for details.

#### B) Model comparison: Resistance to initial inoculation

Among initially infected finches (n = 62), those from the MG-endemic population (VA) had lower loads (i.e., were more resistant) than those from the MG-naive population (AZ; Est = -0.40 ± 0.25). It should be noted that the null model was ranked in the top model set; see Table S9. However, a Kruskal-Wallis rank sum test detected a difference in load among groups categorized by population and sex (X^2^_3_ = 11.04, p = 0.01); see Figure S1. A Dunn test with a Benjamini-Hochberg correction showed this difference existed between female finches from MG-naive and MG-endemic populations, respectively (Z = 3.25, p (adj) = 0.007). This suggests females from the MG-endemic population are more resistant to an initial MG inoculation than females from the MG-naive population.

**Figure S1.** House finch responses to an initial *Mycoplasma gallisepticum* (MG) inoculation. MG loads were assessed 7 days post-inoculation (dppi). Points reflect data from individual birds that were successfully infected; points are jittered for ease of viewing. Data are grouped by host sex (f = female, m = male) and population of origin (az = Arizona, va = Virginia). Y axis is log10 transformed.

C) Model comparison: Eye pathology following initial inoculation

Among finches susceptible to an initial MG inoculation (n = 62), initial eye scores were positively associated with initial load (Est = 0.98 ± 0.21) and were lower in finches from the MG-endemic population (VA) than those from the MG-naive population (AZ; Est = -1.96 ± 0.54); see Figure S2 and Table S10.

**Figure S2.** Eye pathology resulting from experimental *Mycoplasma gallisepticum* infection varies by house finch host population. ‘VA’ indicates finches from Virginia, a relatively MG-endemic population, while ‘AZ’ indicates finches from Arizona, a relatively MG-naive population. Pathology is scored on a three-point scale for each eye and scores for both eyes are combined; the maximum combined scores for individual finches during the first two weeks following inoculation are shown. Data are jittered for easier viewing. Blue represents females and yellow points represent data from individual males.

#### D) Model comparison: Tolerance to initial inoculation

Among finches susceptible to an initial MG inoculation (n = 62), finches from the MG-endemic population (VA) showed higher tolerance than those from the MG-naive population (AZ; Est = 0.13 ± 0.04); see Figure S3 and Table S11.

**Figure S3.** House finches from Virginia (‘VA’) show higher tolerance to an initial *Mycoplasma gallisepticum* inoculation than finches from Arizona (‘AZ’). Shown are tolerance values for finches that were successfully infected following a single MG inoculation (n = 62). Points represent data for individual birds (blue = female) and yellow = male) and are jittered for easier viewing. See methods for further details regarding tolerance quantification.

### Model lists

## A) Susceptibility to the second inoculation (all finches; n = 84)

secinf.null: susceptibility ~ 1

secinf.a: susceptibility ~ second dose

secinf.b: susceptibility ~ second dose + population

secinf.c: susceptibility ~ second dose*population

secinf.d: susceptibility ~ second dose + sex

secinf.e: susceptibility ~ second dose*sex

secinf.f: susceptibility ~ second dose + initial susceptibility

secinf.g: susceptibility ~ second dose*initial susceptibility

secinf.h: susceptibility ~ second dose + initial resistance

secinf.i: susceptibility ~ second dose*initial resistance

secinf.j: susceptibility ~ second dose + initial eye score

secinf.k: susceptibility ~ second dose*initial eye score

secinf.l: susceptibility ~ second dose + population+sex

secinf.m: susceptibility ~ second dose + population*sex

secinf.n: susceptibility ~ second dose + initial susceptibility*population

secinf.o: susceptibility ~ second dose + initial susceptibility + population

secinf.p: susceptibility ~ second dose + initial susceptibility*sex

secinf.q: susceptibility ~ second dose + initial susceptibility + sex

secinf.r: susceptibility ~ second dose + initial eye score*sex

secinf.s: susceptibility ~ second dose + initial eye score + sex

secinf.t: susceptibility ~ second dose + initial eye score*population

secinf.u: susceptibility ~ second dose + initial eye score + population

secinf.v: susceptibility ~ second dose + initial resistance*population

secinf.w: susceptibility ~ second dose + initial resistance + population

secinf.x: susceptibility ~ second dose + initial resistance*sex

secinf.y: susceptibility ~ second dose + initial resistance + sex

## B) Susceptibility to the second inoculation (in finches susceptible to initial infection; n = 37)

secinf2.null: susceptibility ~ 1

secinf2.a: susceptibility ~ second dose

secinf2.b: susceptibility ~ second dose + population

secinf2.c: susceptibility ~ second dose*population

secinf2.d: susceptibility ~ second dose + sex

secinf2.e: susceptibility ~ second dose*sex

secinf2.f: susceptibility ~ second dose + initial resistance

secinf2.g: susceptibility ~ second dose*initial resistance

secinf2.h: susceptibility ~ second dose + initial eye score

secinf2.i: susceptibility ~ second dose*initial eye score

secinf2.j: susceptibility ~ second dose + population + sex

secinf2.k: susceptibility ~ second dose + population*sex

secinf2.l: susceptibility ~ second dose + initial eye score*sex

secinf2.m: susceptibility ~ second dose + initial eye score + sex

secinf2.n: susceptibility ~ second dose + initial eye score*population

secinf2.o: susceptibility ~ second dose + initial eye score + population

secinf2.p: susceptibility ~ second dose + initial resistance*population

secinf2.q: susceptibility ~ second dose + initial resistance + population

secinf2.r: susceptibility ~ second dose + initial resistance*sex

secinf2.s: susceptibility ~ second dose + initial resistance + sex

secinf2.t: susceptibility ~ second dose + initial tolerance

secinf2.u: susceptibility ~ second dose + initial tolerance + population

secinf2.v: susceptibility ~ second dose + initial tolerance + sex

secinf2.w: susceptibility ~ second dose + initial tolerance + sex + population

## C) Resistance to the second inoculation (in all finches susceptible to the second inoculation; n = 22)

secresist.null: resistance ~1

secresist.a: resistance ~ second dose

secresist.b: resistance ~ population

secresist.c: resistance ~ sex

secresist.d: resistance ~ initial susceptibility

secresist.e: resistance ~ initial resistance

secresist.f: resistance ~ initial eye score

secresist.g: resistance ~ second dose + population

secresist.h: resistance ~ second dose + sex

secresist.i: resistance ~ initial eye score + population

secresist.j: resistance ~ second dose + initial susceptibility

secresist.k: resistance ~ second dose + initial resistance

secresist.l: resistance ~ second dose + initial eye score

secresist.m: resistance ~ initial susceptibility + population

secresist.n: resistance ~ initial susceptibility + sex

secresist.o: resistance ~ initial resistance + population

secresist.p: resistance ~ initial resistance + sex

secresist.q: resistance ~ initial eye score + sex

secresist.r: resistance ~ sex + population + second dose + initial resistance

secresist.s: resistance ~ sex + population + second dose + initial eye score

secresist.t: resistance ~ sex + population + second dose + initial susceptibility

## D) Resistance to the second inoculation (in AZ finches susceptible to the second inoculation; n = 13)

secresist.null2: resistance ~ 1

secresist.a2: resistance ~ second dose

secresist.b2: resistance ~ sex

secresist.c2: resistance ~ initial susceptibility

secresist.d2: resistance ~ initial resistance

secresist.e2: resistance ~ initial eye score

## E) Tolerance of the second inoculation (in all finches susceptible to the second inoculation; n = 22)

sectol.null: tolerance ~ 1

sectol.a: tolerance ~ second dose

sectol.b: tolerance ~ population

sectol.c: tolerance ~ sex

sectol.d: tolerance ~ initial susceptibility

sectol.e: tolerance ~ initial resistance

sectol.f: tolerance ~ initial eye score

sectol.g: tolerance ~ second dose + population

sectol.h: tolerance ~ second dose + sex

sectol.i: tolerance ~ initial eye score + population

sectol.j: tolerance ~ second dose + initial susceptibility

sectol.k: tolerance ~ second dose + initial resistance

sectol.l: tolerance ~ second dose + initial eye score

sectol.m: tolerance ~ initial susceptibility + population

sectol.n: tolerance ~ initial susceptibility + sex

sectol.o: tolerance ~ initial resistance + population

sectol.p: tolerance ~ initial resistance + sex

sectol.q: tolerance ~ initial eye score + sex

sectol.r: tolerance ~ sex + population + second dose + initial resistance

sectol.s: tolerance ~ sex + population + second dose + initial eye score

sectol.t: tolerance ~ sex + population + second dose + initial susceptibility

## F) Tolerance of the second inoculation (in AZ finches susceptible to the second inoculation; n = 13)

sectol2.null: tolerance ~ 1

sectol2.a: tolerance ~ second dose

sectol2.b: tolerance ~ sex

sectol2.c: tolerance ~ initial susceptibility

sectol2.d: tolerance ~ initial resistance

sectol2.e: tolerance ~ initial eye score

sectol2.f: tolerance ~ second dose + sex

sectol2.g: tolerance ~ second dose + initial susceptibility

sectol2.h: tolerance ~ second dose + initial resistance

sectol2.i: tolerance ~ second dose + initial eye score

sectol2.j: tolerance ~ initial susceptibility + sex

sectol2.k: tolerance ~ initial resistance + sex

sectol2.l: tolerance ~ initial eye score + sex

## G) Susceptibility to the initial inoculation (all finches*; n = 116)

*Note that this dataset includes some finches that were not included in the central analyses of the paper, which focus on variation in responses to second inoculations; finches that were given a control treatment during the second inoculation phase were therefore excluded from the central analyses.

priminf.null: susceptibility ~1

priminf.a: susceptibility ~ population

priminf.b: susceptibility ~ sex

priminf.c: susceptibility ~ population + sex

priminf.d: susceptibility ~ population*sex

## H) Resistance to the initial inoculation (all finches* susceptible to initial inoculation; n = 62)

*Note that this dataset includes some finches that were not included in the central analyses of the paper, which focus on variation in responses to second inoculations; finches that were given a control treatment during the second inoculation phase were therefore excluded from the central analyses.

primresist.null: resistance ~ 1

primresist.a: resistance ~ population

primresist.b: resistance ~ sex

primresist.c: resistance ~ sex + population

primresist.d: resistance ~ sex*population

## I) Initial inoculation pathology (all finches* susceptible to initial inoculation; n = 62)

*Note that this dataset includes some finches that were not included in the central analyses of the paper, which focus on variation in responses to second inoculations; finches that were given a control treatment during the second inoculation phase were therefore excluded from the central analyses.

glmo0: eye score ~ 1

glmo1: eye score ~ resistance

glmo2: eye score ~ resistance + population

glmo3: eye score ~ resistance*population

glmo4: eye score ~ resistance + sex

glmo5: eye score ~ resistance * sex

glmo6: eye score ~ resistance + sex + population

glmo7: eye score ~ resistance + sex*population

glmo8: eye score ~ resistance*sex + population

## J) Initial inoculation tolerance (all finches* susceptible to initial inoculation; n = 62)

*Note that this dataset includes some finches that were not included in the central analyses of the paper, which focus on variation in responses to second inoculations; finches that were given a control treatment during the second inoculation phase were therefore excluded from the central analyses.

primtol.null: tolerance ~ 1

primtol.a: tolerance ~ population

primtol.b: tolerance ~ sex

primtol.c: tolerance ~ population*sex

primtol.d: tolerance ~ population + sex

### Tables

##### Table S1. Sample sizes of house finches (*Haemorhous mexicanus*) given two *Mycoplasma gallisepticum* (MG) inoculations, by host population of origin (AZ = Arizona, VA = Virginia), second dose concentration, and host sex (n = 84).

| **Host**  **population** | **Second MG dose**  **(CCU/mL)** | **Host**  **sex** | **n** |
| --- | --- | --- | --- |
| AZ | 30 | F | 5 |
| AZ | 30 | M | 6 |
| AZ | 100 | F | 5 |
| AZ | 100 | M | 5 |
| AZ | 300 | F | 3 |
| AZ | 300 | M | 6 |
| AZ | 7000 | F | 7 |
| AZ | 7000 | M | 4 |
| VA | 30 | F | 5 |
| VA | 30 | M | 6 |
| VA | 100 | F | 6 |
| VA | 100 | M | 5 |
| VA | 300 | F | 5 |
| VA | 300 | M | 6 |
| VA | 7000 | F | 4 |
| VA | 7000 | M | 6 |

##### Table S2. Generalized linear models predicting house finch (*Haemorhous mexicanus*; n = 84) susceptibility to a second inoculation of *Mycoplasma gallisepticum* bacteria. All models used a binomial error distribution. Shown are parameter estimates (± standard error) for all models <2 ΔAIC. See Model List A in the Supplement for all model details. Top model is indicated by^§^.

| **Model** | **Intercept** | **Second dose** | **Sex (male)** | **Second dose * Sex (male)** | **Population (VA)** | **Initial resistance** | **k** | **AICc** | **∆AICc** | **Weight** |
| --- | --- | --- | --- | --- | --- | --- | --- | --- | --- | --- |
| secinf.d**^§^** | -2.71 | 4.18^-4^ ± 9.58^-5^ | 1.12 ± 0.65 |  |  |  | 3 | 77.64 | 0.00 | 0.13 |
| secinf.e | -3.43 | 5.72^-4^ ± 1.76^-4^ | 2.04 ± 1.14 | -2.52^-4^ ± 2.11^-4^ |  |  | 4 | 78.28 | 0.64 | 0.10 |
| secinf.l | -2.43 | 4.26^-4^ ± 9.80^-5^ | 1.20 ± 0.67 |  | -0.76 ± 0.62 |  | 4 | 78.29 | 0.65 | 0.10 |
| secinf.a | -1.98 | 3.83^-4^ ± 8.72^-5^ |  |  |  |  | 2 | 78.74 | 1.10 | 0.08 |
| secinf.y | -2.50 | 4.19^-4^ ± 9.65^-5^ | 1.08 ± 0.66 |  |  | -0.11 ± 0.15 | 4 | 79.27 | 1.63 | 0.06 |
| Null | -1.04 |  |  |  |  |  | 1 | 98.66 | 21.02 | 0.00 |

##### Table S3. Generalized linear models predicting house finch (*Haemorhous mexicanus*) susceptibility to a second inoculation of *Mycoplasma gallisepticum* (MG) bacteria. Dataset is restricted to finches successfully infected following an initial MG inoculation (n = 37). All models used a binomial error distribution. Shown are parameter estimates (± standard error) for all models <2 ΔAIC. See Model List B in the Supplement for all model details. Top model is indicated by^§^.

| **Model** | **Intercept** | **Second dose** | **Initial tolerance** | **Sex (male)** | **Population (VA)** | **k** | **AICc** | **∆AICc** | **Weight** |
| --- | --- | --- | --- | --- | --- | --- | --- | --- | --- |
| secinf2.w**^§^** | -3.71 | 5.11^-4^ ± 2.50^-4^ | -12.05 ± 6.55 | 3.61 ± 1.89 | -2.62 ± 1.39 | 5 | 32.60 | 0.00 | 0.35 |
| secinf2.j | -3.02 | 5.08^-4^ ± 2.08^-4^ |  | 2.84 ± 1.48 | -2.77 ± 1.28 | 4 | 34.45 | 1.85 | 0.14 |
| secinf.v | -3.36 | 3.43^-4^ ± 1.86^-4^ | -12.46 ± 5.63 | 2.28 ± 1.39 |  | 4 | 34.57 | 1.97 | 0.13 |
| Null | -1.14 |  |  |  |  | 1 | 43.17 | 10.57 | 0.00 |

##### Table S4. Linear models predicting house finch (*Haemorhous mexicanus*) resistance to a second inoculation of *Mycoplasma gallisepticum* (MG) bacteria. Resistance is quantified as MG load at seven days following the second inoculation (lower loads indicate higher resistance). Dataset is restricted to finches successfully infected following a second MG inoculation (n = 22). Shown are parameter estimates (± standard error) for all models <2 ΔAIC. See Model List C in the Supplement for all model details. Top model is indicated by^§^.

| **Model** | **Intercept** | **Second dose** | **Initial susceptibility**  **(infected)** | **k** | **AICc** | **∆AICc** | **Weight** |
| --- | --- | --- | --- | --- | --- | --- | --- |
| secresist.j**^§^** | 3.91 | 1.73^-4^ ± 6.99^-5^ | -3.02 ± 0.47 | 4 | 72.44 | 0.00 | 0.61 |
| Null | 3.45 |  |  | 2 | 96.20 | 23.76 | 0.00 |

##### Table S5. Linear models predicting house finch (*Haemorhous mexicanus*) resistance to a second inoculation of *Mycoplasma gallisepticum* (MG) bacteria. Resistance is quantified as MG load at seven days following the second inoculation (lower loads indicate higher resistance). Dataset is restricted to finches from Arizona that were successfully infected following a second MG inoculation (n = 13). Shown are parameter estimates (± standard error) for all models <2 ΔAIC. See Model List D in the Supplement for all model details.

| **Model** | **Intercept** | **Initial susceptibility (infected)** | **Initial load** | **Initial  eye score** | **k** | **AICc** | **∆AICc** | **Weight** |
| --- | --- | --- | --- | --- | --- | --- | --- | --- |
| secresist.c2 | 4.77 | -2.75 ± 0.74 |  |  | 3 | 50.88 | 0.00 | 0.41 |
| secresist.d2 | 4.31 |  | -0.64 ± 0.17 |  | 3 | 51.02 | 0.13 | 0.38 |
| secresist.e2 | 4.29 |  |  | -1.09 ± 0.33 | 3 | 52.49 | 1.61 | 0.18 |
| Null | 3.29 |  |  |  | 2 | 57.96 | 7.08 | 0.01 |

##### Table S6. Linear models predicting house finch (*Haemorhous mexicanus*) tolerance to a second inoculation of *Mycoplasma gallisepticum* (MG) bacteria. Tolerance is quantified as residuals of a generalized linear model (binomial family) using MG load at seven days post-inoculation to predict maximum eye score recorded over the first two weeks following inoculation. Analysis is restricted to data from finches successfully infected following a second inoculation (n = 22). Residuals of this model, multiplied by -1, were used as tolerance values; thus, larger values indicate higher tolerance (i.e., relatively less pathology per unit pathogen load relative to the rest of the population). Shown are parameter estimates (± standard error) for all models <2 ΔAIC. See Model List E in the Supplement for all model details.

| **Model** | **Intercept** | **Second dose** | **Population (VA)** | **Initial susceptibility (infected)** | **k** | **AICc** | **∆AICc** | **Weight** |
| --- | --- | --- | --- | --- | --- | --- | --- | --- |
| Null | 2.22^-13^ |  |  |  | 2 | 4.57 | 0.00 | 0.21 |
| sectol.a | -7.83^-2^ | 1.74^-5^ ± 1.59^-5^ |  |  | 3 | 6.00 | 1.44 | 0.10 |
| sectol.b | -0.04 |  | 0.10 ± 0.11 |  | 3 | 6.34 | 1.77 | 0.09 |
| sectol.d | -0.04 |  |  | 0.10 ± 0.11 | 3 | 6.40 | 1.83 | 0.08 |

##### Table S7. Linear models predicting house finch (*Haemorhous mexicanus*) tolerance to a second inoculation of *Mycoplasma gallisepticum* (MG) bacteria. Tolerance is quantified as residuals of a generalized linear model (binomial family) using MG load at seven days post-inoculation to predict maximum eye score recorded over the first two weeks following inoculation. Residuals of this model, multiplied by -1, were used as tolerance values; thus, larger values indicate higher tolerance (i.e., relatively less pathology per unit pathogen load relative to the rest of the population). Dataset is restricted to finches from Arizona that were successfully infected following a second MG inoculation (n = 13). Shown are parameter estimates (± standard error) for all models <2 ΔAIC. See Model List E in the Supplement for all model details. Top model is indicated by^§^.

| **Model** | **Intercept** | **Initial susceptibility (infected)** | **Second dose** | **k** | **AICc** | **∆AICc** | **Weight** |
| --- | --- | --- | --- | --- | --- | --- | --- |
| sectol2.g^§^ | -3.89^-2^ | 0.33 ± 0.12 | 3.86^-5^ ± 1.82^-5^ | 4 | 6.79 | 0.00 | 0.30 |
| sectol2.c | -0.20 | 0.30 ± 0.14 |  | 3 | 7.29 | 0.50 | 0.24 |
| Null | -0.04 |  |  | 2 | 8.53 | 1.74 | 0.13 |

##### Table S8. Generalized linear models predicting house finch (*Haemorhous mexicanus*; n = 116) susceptibility to an initial inoculation of *Mycoplasma gallisepticum* (MG) bacteria. All models use a binomial family. Shown are parameter estimates (± standard error) for all models <2 ΔAIC. See Model List G in the Supplement for all model details.

| **Model** | **Intercept** | **Sex (M)** | **Population (VA)** | **k** | **AICc** | **∆AICc** | **Weight** |
| --- | --- | --- | --- | --- | --- | --- | --- |
| Null | 0.14 |  |  | 1 | 162.29 | 0.00 | 0.43 |
| priminf.b | 0.31 | -0.34 ± 0.38 |  | 2 | 163.52 | 1.23 | 0.24 |
| priminf.a | 0.25 |  | -0.25 ± 0.37 | 2 | 163.91 | 1.62 | 0.19 |

##### Table S9. Generalized linear models predicting house finch (*Haemorhous mexicanus*) resistance to an initial inoculation of *Mycoplasma gallisepticum* (MG) bacteria. Resistance is quantified as MG load at seven days following the second inoculation (lower loads indicate higher resistance). All models use a negative binomial family. Data are restricted to finches that were successfully infected following initial inoculation (n = 62). Shown are parameter estimates (± standard error) for all models <2 ΔAIC. See Model List H in the Supplement for all model details. Top model is indicated by^§^.

| **Model** | **Intercept** | **Population (VA)** | **Sex (M)** | **Sex (M) * population (VA)** | **k** | **AICc** | **∆AICc** | **Weight** |
| --- | --- | --- | --- | --- | --- | --- | --- | --- |
| primresist.a^§^ | 10.90 | -0.40 ± 0.25 |  |  | 3 | 1378.41 | 0.00 | 0.33 |
| Null | 10.75 |  |  |  | 2 | 1378.81 | 0.40 | 0.27 |
| primresist.d | 11.10 | -0.79 ± 0.34 | -0.50 ± 0.32 | 0.81 ± 0.50 | 5 | 1379.93 | 1.52 | 0.16 |
| primresist.c | 10.98 | -0.43 ± 0.25 | -0.17 ± 0.25 |  | 4 | 1380.22 | 1.81 | 0.14 |

##### Table S10. Ordinal regression models predicting house finch (*Haemorhous mexicanus*) pathology following an initial inoculation of *Mycoplasma gallisepticum* (MG) bacteria. Maximum initial eye score is the highest eye score recorded within the first two weeks following initial inoculation. Data are restricted to finches that were successfully infected following initial inoculation (n = 62). Shown are parameter estimates (± standard error) for all models <2 ΔAIC. Full models and parameters available in code. See Model List I in the Supplement for all model details. Top model is indicated by^§^.

| **Model** | **0\| 0.5** | **0.5\| 1** | **1\| 1.5** | **1.5\| 2** | **2\| 2.5** | **2.5\| 3** | **3\| 5** | **5\| 6** | **Population (VA)** | **Initial load** | **Initial load * population (VA)** | **k** | **AICc** | **∆AICc** | **Weight** |
| --- | --- | --- | --- | --- | --- | --- | --- | --- | --- | --- | --- | --- | --- | --- | --- |
| glmo2^§^ | -0.21 | 1.42 | 2.83 | 3.60 | 4.56 | 5.01 | 6.90 | 8.09 | -1.96 ± 0.54 | 0.98 ± 0.21 |  | 10 | 231.16 | 0.00 | 0.50 |
| glmo3 | -8.50^-3^ | 1.59 | 3.00 | 3.77 | 4.75 | 5.20 | 7.10 | 8.29 | -1.31 ± 1.62 | 1.03 ± 0.24 | -0.18 ± 0.42 | 11 | 233.07 | 1.90 | 0.19 |
| Null | -2.06 | -0.97 | -0.13 | 0.33 | 0.97 | 1.33 | 2.98 | 4.11 |  |  |  | 8 | 268.39 | 37.22 | 0.00 |

##### Table S11. Linear models predicting house finch (*Haemorhous mexicanus*) tolerance to an initial inoculation of *Mycoplasma gallisepticum* (MG) bacteria. Tolerance is quantified as residuals of a generalized linear model (binomial family) using MG load at seven days post-inoculation to predict maximum eye score recorded over the first two weeks following inoculation. Residuals of this model, multiplied by -1, were used as tolerance values; thus, larger values indicate higher tolerance (i.e., relatively less pathology per unit pathogen load relative to the rest of the population). Data are restricted to finches that were successfully infected following initial inoculation (n = 62). Shown are parameter estimates (± standard error) for all models <2 ΔAIC. See Model List J in the Supplement for all model details. Top model is indicated by^§^.

| **Model** | **Intercept** | **Population (VA)** | **Sex (M)** | **k** | **AICc** | **∆AICc** | **Weight** |
| --- | --- | --- | --- | --- | --- | --- | --- |
| primtol.a^§^ | -0.05 | 0.13 ± 0.04 |  | 3 | -45.20 | 0.00 | 0.61 |
| primtol.d | -0.07 | 0.13 ± 0.04 | 0.03 ± 0.04 | 4 | -43.54 | 1.65 | 0.27 |
| Null | 6.19^-16^ |  |  | 2 | -38.63 | 6.56 | 0.02 |
